# Subject classification and cross-time prediction based on functional connectivity and white matter microstructure features in a rat model of Alzheimer’s using machine learning

**DOI:** 10.1186/s13195-023-01328-0

**Published:** 2023-11-07

**Authors:** Yujian Diao, Bernard Lanz, Ileana Ozana Jelescu

**Affiliations:** 1 Animal Imaging and Technology Section, CIBM Center for Biomedical Imaging, Ecole Polytechnique Fédérale de Lausanne, Lausanne, Switzerland; 2 Laboratory for Functional and Metabolic Imaging, Ecole Polytechnique Fédérale de Lausanne, Lausanne, Switzerland; 3 Department of Radiology, Lausanne University Hospital and University of Lausanne, Lausanne, Switzerland

**Keywords:** Animal model of Alzheimer’s disease, Intracerebroventricular streptozotocin, Classification, Diffusion MRI, Microstructure, White matter, Functional connectivity, Logistic regression, Machine learning

## Abstract

**Background:**

The pathological process of Alzheimer’s disease (AD) typically takes decades from onset to clinical symptoms. Early brain changes in AD include MRI-measurable features such as altered functional connectivity (FC) and white matter degeneration. The ability of these features to discriminate between subjects without a diagnosis, or their prognostic value, is however not established.

**Methods:**

The main trigger mechanism of AD is still debated, although impaired brain glucose metabolism is taking an increasingly central role. Here, we used a rat model of sporadic AD, based on impaired brain glucose metabolism induced by an intracerebroventricular injection of streptozotocin (STZ). We characterized alterations in FC and white matter microstructure longitudinally using functional and diffusion MRI. Those MRI-derived measures were used to classify STZ from control rats using machine learning, and the importance of each individual measure was quantified using explainable artificial intelligence methods.

**Results:**

Overall, combining all the FC and white matter metrics in an ensemble way was the best strategy to discriminate STZ rats, with a consistent accuracy over 0.85. However, the best accuracy early on was achieved using white matter microstructure features, and later on using FC. This suggests that consistent damage in white matter in the STZ group might precede FC. For cross-timepoint prediction, microstructure features also had the highest performance while, in contrast, that of FC was reduced by its dynamic pattern which shifted from early hyperconnectivity to late hypoconnectivity.

**Conclusions:**

Our study highlights the MRI-derived measures that best discriminate STZ vs control rats early in the course of the disease, with potential translation to humans.

**Supplementary Information:**

The online version contains supplementary material available at 10.1186/s13195-023-01328-0.

## Background

Alzheimer’s disease (AD) as a progressive neurodegenerative disorder is the main cause of dementia, which is characterized by a decline in cognitive functions such as thinking, remembering, and reasoning. AD can be divided into two major categories: sporadic AD and familial AD. The familial AD that accounts for less than 5% of all AD cases [9] is usually caused by a genetic mutation, whereas sporadic AD accounting for the majority of AD cases is multifactorial [18]. Pathologically, AD is characterized by extracellular deposits of Aβ peptides as senile plaques, intraneuronal neurofibrillary tangles, reduced brain glucose metabolism and large-scale neuronal loss in the most affected regions of the brain, such as the medial temporal lobe and neocortical structures [13, 23, 31, 67, 68].

Non-invasive brain imaging techniques such as magnetic resonance imaging (MRI) play a vital role in detecting early changes in the brain associated with AD. Gross cerebral atrophy [37], white matter (WM) degeneration [3, 19, 20, 29, 51] and altered functional connectivity (FC) [2, 12, 15, 34, 39] were found to be relevant biomarkers. Recently, resting-state FC has been proposed to identify individuals at risk for Alzheimer’s disease in the early stages [45, 110, 112]. The characterization of the temporal progression of microstructural and FC changes promises to provide an understanding of disease mechanisms, an effective disease staging, and a window for therapeutic intervention.

As the pathological cascade of AD takes up to years or even decades from the dementia onset to full-blown manifestations, it remains challenging to acquire comprehensive longitudinal data on prospective AD subjects. As an alternative, animal models can be valuable tools to obtain data across the lifespan and study each of the contributors to the AD cascade individually, thus untangling direct effects of contributors and their interactions. Although numerous animal models have been developed to replicate the AD phenotype, most of them are transgenic models which are less representative of sporadic AD and are primarily based on the Aβ hypothesis [16, 59], which is increasingly challenged [56]. However, with glucose hypometabolism being increasingly recognized as a potential cause of AD [21, 46, 62], animal models of brain insulin resistance have been developed by an intracerebroventricular (icv) injection of streptozotocin (STZ) [60, 61, 91]. The icv-STZ animals have been reported to manifest typical pathological features of AD such as extracellular accumulation of Aβ, tau hyperphosphorylation, neuronal loss, axonal damage, and demyelination in the hippocampus and fimbria [30, 60, 91, 97], reduced glucose uptake [43, 97] and oxidative stress [66, 91], without developing systemic diabetes. From a behavioral perspective, STZ rats demonstrate lower post-shock latency time in the passive avoidance test [60], higher escape latency in the elevated plus maze, shorter exploration time of the novel arm in the Y-maze, poorer object recognition and tone fear memory [75, 95], all pointing to impaired short-term memory.

In a previous work, we performed a comprehensive longitudinal study [97] in an icv-STZ rat model to quantitatively characterize alterations in FC and in WM microstructure using resting-state functional MRI (fMRI) and advanced diffusion MRI techniques, respectively, as well as in brain glucose uptake captured by ^18^FDG-PET. By comparing the STZ group to the control group, non-invasive MRI-derived measures of functional breakdown and WM degeneration were identified and evaluated in the context of brain glucose hypometabolism. Alterations in resting-state FC in STZ rats were found in brain regions closely associated with AD [2, 15] with broadly increased then decreased connectivity at early and late timepoints, respectively. WM microstructure metrics derived from DKI (an extension of diffusion tensor imaging (DTI) that provides complementary information about tissue heterogeneity [53]) and the WMTI-Watson biophysical model [32, 54] revealed specifically intra-axonal damage and axonal loss in the corpus callosum, fimbria and cingulum of STZ rats. The temporal dynamics of both WM integrity and FC were consistent with previously reported nonmonotonic trajectories of brain alterations along AD progression in humans [26, 29, 84, 88]. These findings not only reinforced the suitability of the icv-STZ animal model for sporadic AD but also proposed MRI-derived features to identify alterations in the prodromal stage and monitor disease progression.

In this study, we go beyond descriptive statistics and evaluate the microstructural and functional measures for their potential to discriminate between control and STZ groups at a given timepoint and across time. The data used is the current analysis is more extensive than the ones underlying the group difference analysis in [97] through the addition of a fourth longitudinal timepoint and the inclusion of more animals, in particular for FDG-PET, to better evaluate regional brain glucose metabolism in the icv-STZ rats. We utilize quantitative MRI measures as features using machine learning (ML) to train classification models such as logistic regression (LR) to differentiate individual subjects. Moreover, we employ explainable artificial intelligence methods to interpret ML model outcomes. For example, the importance of each feature in terms of the absolute value of LR coefficients is used to identify features best discriminating STZ rats from controls. SHAP values (SHapley Additive exPlanations) [69], a model-agnostic approach, are used to interpret the model outcomes and to improve model transparency. Finally, the dynamic relationships between the functional and microstructural measures in STZ rats are highlighted at the early and late timepoints of disease progression. In a nutshell, our study highlights the MRI-derived measures that best discriminate STZ vs control rats at various stages of the disease, with potential translation to humans.

## Methods

### Study design

**Fig. 1:**
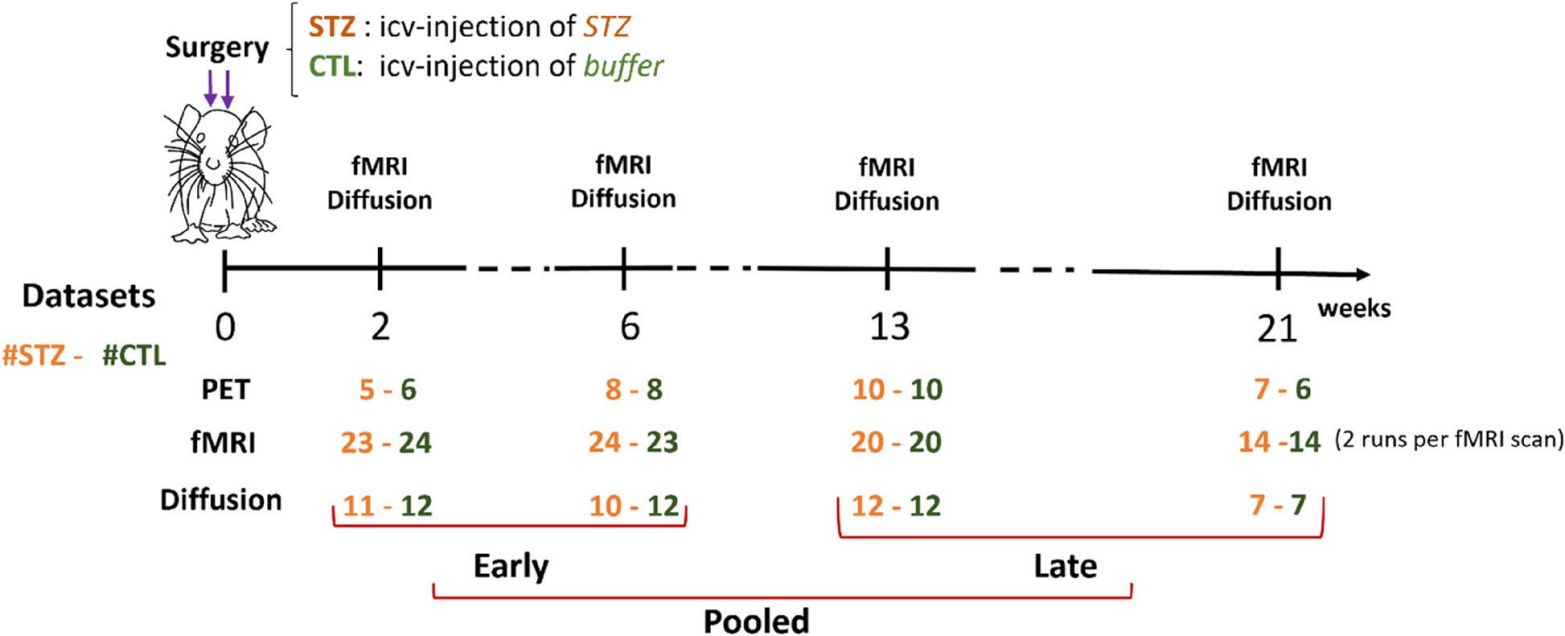
Experimental timeline. MRI: fMRI and diffusion MRI data were collected at 2, 6, 13, and 21 weeks after icv-STZ injection. *N* = 24 rats were included in total (12 STZ / 12 CTL) as reflected in the diffusion MRI datasets at 13 weeks. A lower number of datasets at other timepoints are due to poor data quality (2, 6 weeks) or missing datasets at 21 weeks due to an MRI system upgrade. For fMRI, two runs per rat were acquired for each MRI session which increased the number of datasets. The 4 timepoints were further grouped into *early* and *late* time groups and finally the *pooled* dataset. Sample sizes (STZ/CTL) in the 3 datasets for fMRI and diffusion MRI are as follows. Early: 47/47 (fMRI), 21/24 (diffusion); late: 34/34 (fMRI), 19/19 (diffusion); pooled: 81/81 (fMRI), 40/43 (diffusion). PET: FDG-PET data were also collected at the four timepoints in a subset of rats (*N* = 20 total) and were used to assess group differences in regional brain glucose uptake. Dataset numbers at each timepoint vary due to PET scanner unavailability (especially fewer datasets at 2 weeks) or missing MRI at 21 weeks which also prompted dropping the PET scan acquisition

Male Wistar rats (236 ± 11 g) underwent a bilateral icv-injection of either streptozotocin (3 mg/kg, STZ group) or buffer (CTL group) as previously described [97]. When delivered exclusively to the brain, streptozotocin induces impaired brain glucose metabolism and is used as a model of sporadic AD [40, 66]. Resting-state fMRI, diffusion MRI, and FDG-PET data were acquired longitudinally at four timepoints (2, 6, 13, and 21 weeks since icv injection) (Fig. 1). Timepoints were chosen to be consistent with previous rat STZ studies while also accommodating for constraints of repeated MRI scanning related to anesthesia, cannulations, and scanner availability.

### MRI data acquisition

Animals were initially anesthetized using isoflurane (4% for induction and 1–2% for maintenance in an oxygen/air mixture of 30%/70%) and positioned in a homemade MRI cradle equipped with a fixation system (bite bar and ear bars). A catheter was inserted subcutaneously on the back of the animal for later medetomidine delivery. One hour before starting the resting-state fMRI acquisition, anesthesia was switched from isoflurane to medetomidine (Dorbene, Graeub, Switzerland), which preserves neural activity and vascular response better than isoflurane [83, 104], with an initial bolus of 0.1 mg/kg followed by a continuous perfusion of 0.1 mg/kg/h [85]. The commercial solution at 1 mg/mL was diluted to 0.033 mg/mL. Throughout the experiment, the breathing rate was monitored using a respiration pillow and a rectal thermometer, respectively. Body temperature was maintained around (37 ± 0.5) °C. The breathing rate under medetomidine was around 85 bpm. At the end of the scanning session, animals were woken up with an intramuscular injection of atipamezole (Alzane, Graeub, Switzerland) at 0.5 mg/kg.

MRI experiments were conducted on a 14.1 T small animal scanner. As a result of the system upgrade, data were acquired with two different consoles for the magnet: Varian system (Varian Inc.) equipped with 400 mT/m gradients (cohort 1, *N* = 17 rats) and Bruker system (Ettlingen, Germany) equipped with 1 T/m gradients (cohort 2, *N* = 7 rats), both using the same in-house built quadrature surface transceiver. The acquisition parameters were the same for the two cohorts. Each cohort comprised animals from both groups: cohort 1 (CTL/STZ, *N* = 8/9 rats) and cohort 2 (CTL/STZ, *N* = 4/3 rats).

Structural ${T}_{2}$-weighted images were collected using a fast spin-echo sequence with the following parameters: TE/TR = 10.17/3000 ms, echo train length: 4, matrix size = 128 × 128, FOV = 19.2 × 19.2 mm^2^, voxel size = 0.15 × 0.15 mm^2^, 30 coronal 0.5-mm slices, scan time = 10 min.

Diffusion-weighted data were acquired using a pulsed-gradient spin-echo segmented echo-planar-imaging (EPI) sequence, with the following protocol: 4 b = 0 images and 3 shells at b = 0.8/1.3/2.0 ms/µm^2^, with 12, 16 and 30 directions, respectively; δ/Δ = 4/27 ms; TE/TR = 48/2500 ms; 4 shots; matrix size = 128 × 64, field-of-view = 23 × 17 mm^2^, voxel size = 0.18 × 0.27 mm^2^, 9 coronal 1-mm slices, 4 repetitions, scan time = 1 h.

Resting-state fMRI data were acquired using a two-shot gradient-echo EPI sequence as follows: TE/TR = 10/800 ms, TR_volume_ = 1.6 s, matrix size = 64 × 64, field-of-view = 23 × 23 mm^2^, voxel size = 0.36 × 0.36 mm^2^ and 8 coronal 1.12-mm slices, 370 repetitions, scan time = 10 min. Two fMRI runs were acquired in each MRI session.

It should be noted that the phrase “resting-state fMRI” refers to the fact that the fMRI acquisition was performed during an idle state of the rat, as opposed to “task fMRI” which would present the animals with a sensory stimulation paradigm for example. Nonetheless, all animals were in fact anesthetized using medetomidine, which does alter brain activity as compared to awake animals. While awake rodent fMRI is a promising lead in the field, anesthetized fMRI is still the norm in rodent experiments [38].

### FDG-PET data acquisition

All procedures are identical to those described in [97], where more details can be found. Briefly, rats housed with free access to food and water were anesthetized using isoflurane (2% for induction) for tail vein cannulation for tracer delivery, and subsequently transferred on a temperature-regulated PET scanner bed. Within the first minute of PET acquisition, a bolus of roughly 50 MBq ^18^F-FDG (Advanced Accelerator Applications, Geneva, Switzerland) in 50–300 µL was manually injected through the tail vein and followed by a saline chase.

All PET experiments were performed on an avalanche photodiode-based LabPET-4 small-animal scanner (Gamma Medica-Ideas Inc.) as described in [63]. Briefly, data were collected in list-mode and images of the labeling steady-state were reconstructed from coincidences between 30 and 50 min after tracer injection using the built-in maximum likelihood expectation maximization (MLEM) iterative reconstruction algorithm (30 iterations) with a circular field of view (FOV) of 80 mm. The reconstructed voxel size was 0.25 × 0.25 × 1.18 mm. Steady-state radioactivity density images were then normalized for the effective injected FDG dose and the animal weight to generate standardized uptake value (SUV) maps.

### Data processing

FMRI data processing followed the PIRACY pipeline [25] which included denoising [100], susceptibility distortion correction [94], slice-timing correction [42], spatial smoothing, and removal of physiological noise following independent component (IC) analysis decomposition. FC matrices between 28 regions of interest (ROIs) based on the Waxholm Space Atlas were computed, co-varying for the global signal [25]. Statistical comparisons of FC between the STZ and CTL groups at each timepoint were performed using NBS [109] to identify network connections that showed significant between-group differences. Specifically, NBS uses one-tailed two-sample *t*-test to detect differences in group averaged FC between the two groups. Thereby, two contrasts (STZ > CTL and STZ < CTL) were tested separately. A *t*-statistic threshold of 2.2 was chosen on the basis of medium-to-large sizes of the subnetwork comprised connections with their *t*-statistic above the threshold [98] as well as the underlying *p*-values. Significance (*p* ≤ 0.05) was tested after family-wise error rate correction using non-parametric permutation (*N* = 5000).

**Fig. 2:**
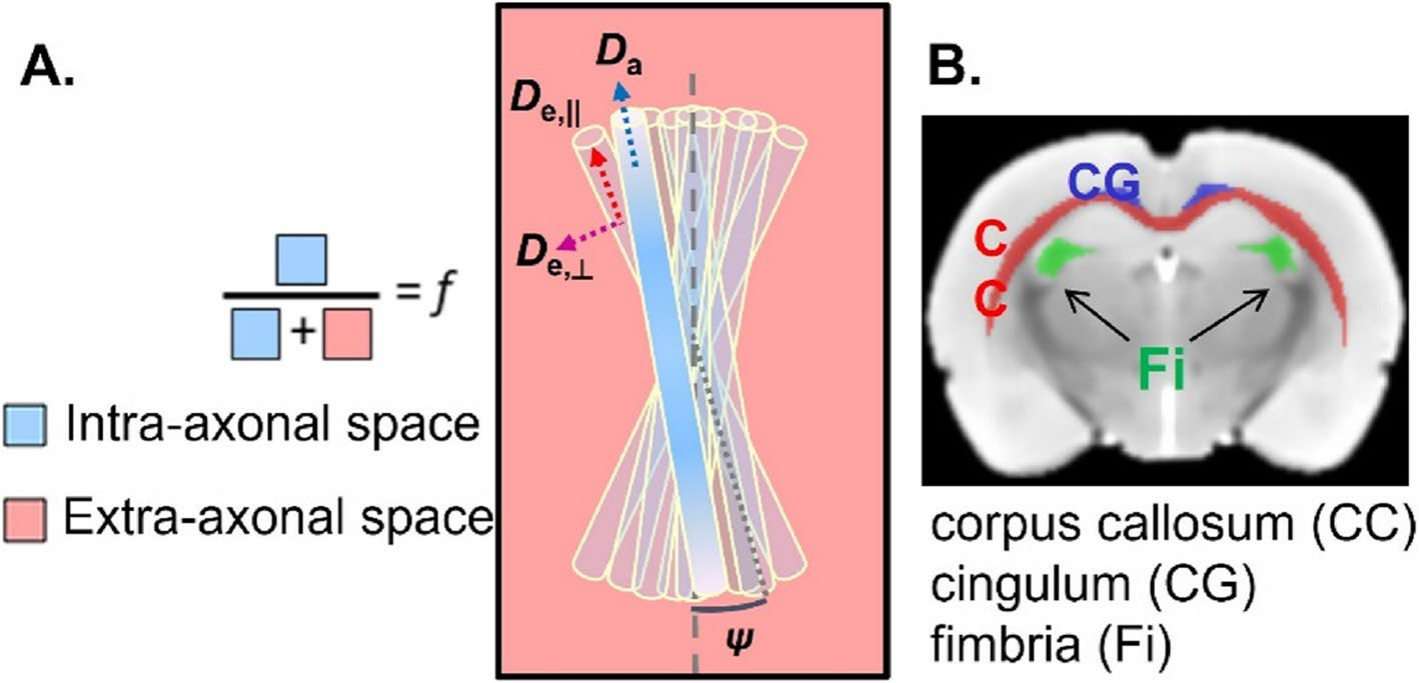
**A** Schematic of the WMTI-Watson biophysical model. The diffusion signal is described in terms of two non-exchanging compartments, the intra and extra-axonal spaces. Here, the axons are modeled as sticks with a radius equal to zero. The intra-axonal space is described by a relative volume fraction of water *f* and by the parallel intra-axonal diffusivity ${D}_{a}$. The perpendicular intra-axonal diffusivity is negligible at the relevant diffusion times and weightings. The bundle of axons is embedded in the extra-axonal space, characterized by its parallel ${D}_{e, \parallel }$ and perpendicular extra-axonal diffusivities ${D}_{e,\perp }$. The axons’ orientations are modeled by a Watson distribution, which is characterized by $\langle {(\mathit{cos}\psi )}^{2}\rangle \equiv {c}_{2}$. **B** The white matter ROIs

Diffusion data processing included MP-PCA denoising [100], Gibbs-ringing correction [57], and correction for susceptibility distortions and eddy currents using FSL’s eddy [5]. The diffusion and kurtosis tensors were estimated using a weighted linear least squares algorithm [101], and typical DTI and DKI-derived metrics were computed: fractional anisotropy (FA), mean/axial/radial diffusivity and mean/axial/radial kurtosis. The DTI diffusivities correspond to the average overall diffusivity in the voxel, along the main orientation of the WM bundle (AD), perpendicular to that (RD), and averaged over all directions (MD). Kurtosis is a clinically feasible extension of DTI that also estimates the non-Gaussian nature of diffusion in the tissue, and is thus a measure of heterogeneity or variance in the diffusion properties of the water molecules at the voxel level (AK, RK, MK). The biophysical WMTI-Watson model [54] (Fig. 2) was estimated voxel-wise in WM regions using nonlinear least squares fitting to extract its microstructure parameters: axonal water fraction $f$, a proxy for axonal density; intra-axonal diffusivity ${D}_{a}$, a proxy for the crowding of the intra-axonal space and thus for axon integrity; extra-axonal parallel and perpendicular diffusivities ${D}_{e, \parallel }, {D}_{e,\perp }$, sensitive to myelination, packing, and cell crowding in the extra-axonal space; and axon’s orientation coherence within the WM bundle ${c}_{2}$, where 1 corresponds to axons perfectly parallel to each other and 1/3 to isotropically distributed axons (without a preferential orientation of the bundle). In parallel, fractional anisotropy (FA) maps were registered to an FA template in the Waxholm Space using linear and non-linear registration in FSL [52] and the corpus callosum (CC), cingulum (CG) and fimbria (Fi) of the hippocampus were automatically segmented. For each ROI, average tensor and biophysical model metrics were calculated. Group differences were tested using *t*-test.

FDG-PET steady-state SUV maps were registered to their corresponding T_2_-weighted anatomical MR images with cross-correlation using ANTs [7, 8], which was in turn registered to the Waxholm Space Atlas of the rat brain (https://www.nitrc.org/projects/whs-sd-atlas) using linear and non-linear registration [8] and 26 ROIs were automatically segmented. SUV images were normalized by the mean SUV over the brain to obtain SUVr maps corrected for inter-rat experimental variability [44]. Regional differences in SUVr between STZ and CTL groups were evaluated at each timepoint using a one-tailed Mann–Whitney *U*-test (STZ < CTL), at a significance level of α = 0.05.

### Classification using logistic regression

For FC-based classification, correlation coefficients between ROIs in the FC matrix were taken as classification features by vectorizing the upper triangle of the FC matrix since FC is symmetric. To study the connection between statistical differences and classification performance in discriminating the two groups, significant edges from the NBS analysis were selected as a reduced list of features for classification. Datasets were grouped as early (2 and 6 weeks, *N* = 94) and late timepoints (13 and 21 weeks, *N* = 68), as well as all timepoints (pooled, *N* = 162). At each timepoint, the number of available samples was relatively small, which can pose challenges in building robust machine learning models. By merging data from two or four time points, we aimed to enhance the dataset size, thereby improving the model's ability to generalize and make reliable predictions. Furthermore, the datasets combined from distinct timepoints are not purely duplicates, and they exhibit inherent variabilities due to disease progression and MRI inter-run variability. STZ/CTL classification using a LR model was trained and tested on each subset (pooled, early, and late), which was normalized to [-1, 1] and randomly split into training (70%) and test datasets (30%). Since the data size was relatively small, the procedure of data splitting, training, and testing was repeated 1000 times, and results were aggregated in order to reduce bias.

For microstructure-based classification, there were two types of features for each of the three WM ROIs: I) DKI tensor metrics including FA, axial, mean, and radial diffusivities (AxD, MD, RD), axial, mean, and radial kurtosis (AK, MK, RK); II) WMTI-Watson model parameters including *f*, ${D}_{a}$, ${D}_{e, \parallel }$, ${D}_{e,\perp }$, and ${c}_{2}$ (Fig. 2). These two kinds of features were used in two ways: as independent feature sets (i.e., DKI only and WMTI only) and combined as a single feature set. As for FC, diffusion datasets were grouped as early (2 and 6 weeks, *N* = 45), late (13 and 21 weeks, *N* = 38), and all timepoints (pooled, *N* = 83). LR models of STZ/CTL classification were trained and tested on the three datasets independently with 70% data for training and 30% for testing. The procedure was also repeated 1000 times.

Considering the small data size, feature dimensionality reduction was also tried for each classification by employing the principal component analysis (PCA) with various numbers of components.

Moreover, we tested classifying STZ and CTL rats by combining the FC and WM microstructure metrics in two distinct ways. One was to create a single classifier based on the concatenation of features of FC and microstructure metrics. The second way was using ensemble learning [81, 87] where three independent classifiers were built each based on one of the three types of features (FC, DKI, and WMTI). Their predictions for each class were aggregated and the class with the majority vote was retained. Datasets for which both FC and dMRI were not available jointly (e.g., as a result of partial or artefacted data) were removed, resulting in a slightly reduced sample size (STZ/CTL = 38/41, instead of 40/43 possible datasets across both groups and timepoints).

Finally, cross prediction was performed, which means a classifier was trained on the dataset of one timepoint (e.g., early) and tested on the other timepoint (e.g., late) and vice versa. Cross prediction was tested on classifiers built on both separate and joint features.

### Model explainability and feature importance

Classification accuracy was used to assess the performance of a LR model in classifying STZ and CTL rats. However, to better interpret and explain the model outcome, we further calculated the importance of each feature in driving a model to predict the STZ class in terms of the absolute values of LR coefficients [93]. The mean feature importance was computed by averaging the absolute LR coefficient of each feature over the 1000 repetitions of training/test data splits, along with mean classification accuracy and standard deviation.

SHAP values that have been widely used for interpreting ML models were also calculated. In this study, SHAP values were computed for different types of features (i.e., significant FC connections, DKI metrics, or WMTI parameters) at each of the three timepoints (early, late, and pooled) to measure the individual impact of each feature on the model outcome. With the combination of classification accuracy and SHAP values, we were able to validate each measure’s ability to discriminate STZ rats from controls. As SHAP values are instance-based, they cannot be averaged over repeated training scenarios like the LR coefficient. Instead, we selected representative SHAP value sets from the 1000 candidates by choosing the ones that had relatively high classification accuracies (> 0.9) in both training and test datasets such that the model would have good performance as well as high generalizability.

## Results

### FDG uptake differences

**Fig. 3:**
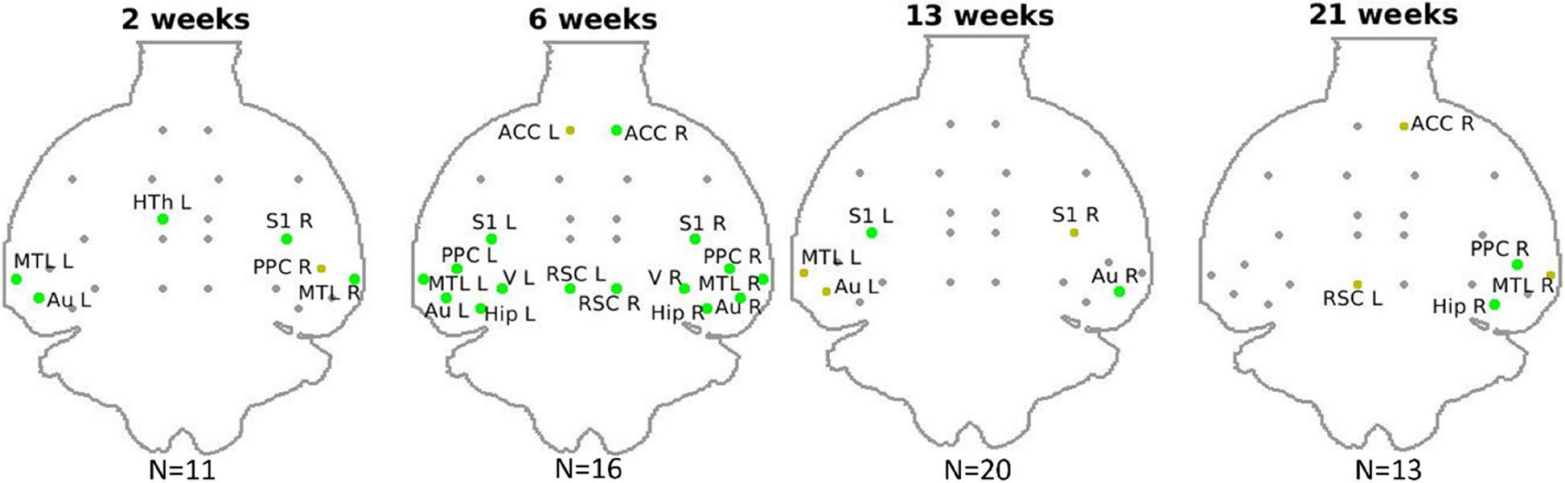
Group differences in SUVr at each timepoint. Green: ROIs with significantly lower SUVr in STZ (*p* < 0.05 using one-tailed Mann–Whitney *U* test, STZ < CTL). Dark yellow: trend of lower SUVr (*p* < 0.1). Correction for multiple comparisons was not applied given the small number of animals per group. ACC, anterior cingulate cortex; RSC, retrosplenial cortex; PPC, posterior parietal cortex; MTL, medial temporal lobe; Hip, hippocampus; Sub, subiculum; Au, auditory; V, visual; S1/S2, primary/secondary somatosensory; M, motor cortices; Str, striatum; Tha, thalamus; HTh, hypothalamus; L/R, left/right

The SUVr in STZ rats was reduced in multiple brain regions as compared to CTL, confirming the locally impaired glucose metabolism (Fig. 3). Glucose hypometabolism concerned mainly DMN and LCN regions. Differences were present across time, with the most widespread changes occurring at 6 weeks after icv injection.

### FC-based classification

**Fig. 4:**
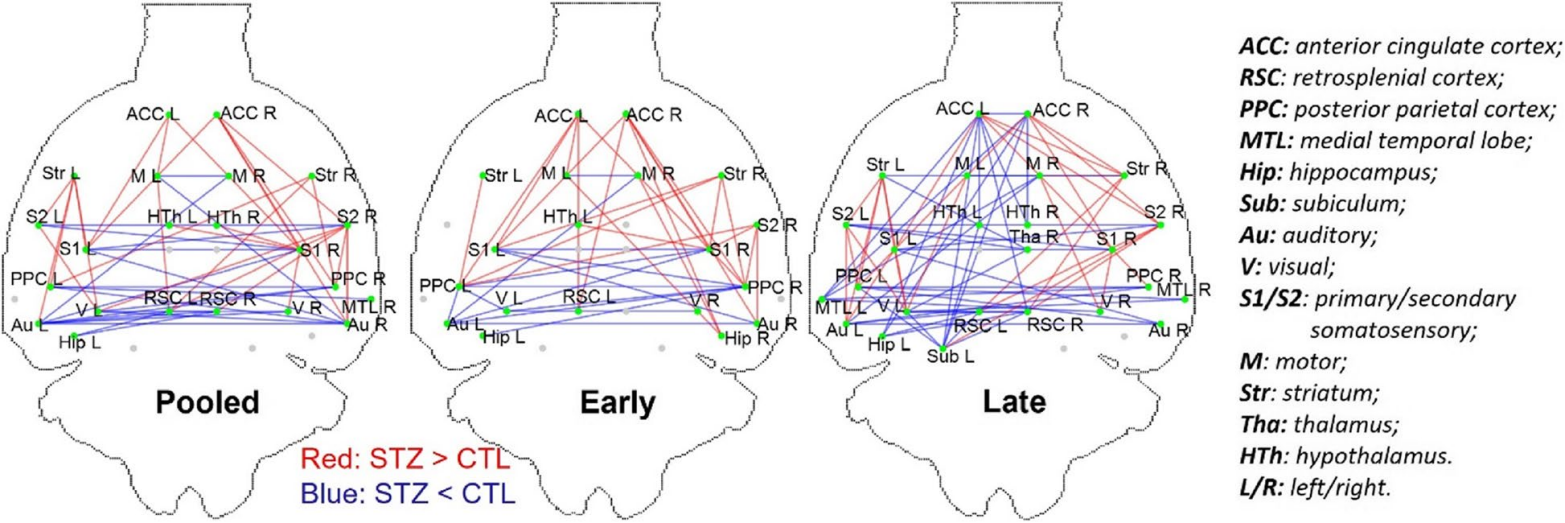
Graph networks of significant group difference using NBS with *p* < 0.05 (family-wise error rate corrected) for the 3 datasets (Pooled, Early and Late). Blue/red edges represent edges where STZ rats have weaker/stronger FC than CTL

In Fig. 4, graph networks highlight the group differences in nodal connections in the pooled, early, and late datasets. Up to 6 weeks after icv injection (early), the STZ group displayed increased connectivity within the default mode network (DMN) (including the anterior cingulate cortex (ACC), retrosplenial cortex (RSC), hippocampus and subiculum) as well as striatum, and decreased connectivity between the DMN (RSC, posterior parietal cortex (PPC) and hippocampus) and the lateral cortical network including primary and secondary somatosentory cortex (S1, S2) and the motor cortex, as compared to CTL rats. From 13 weeks on (late), reduced connectivity became more widespread within the DMN and lateral cortical network in STZ rats.

**Fig. 5:**
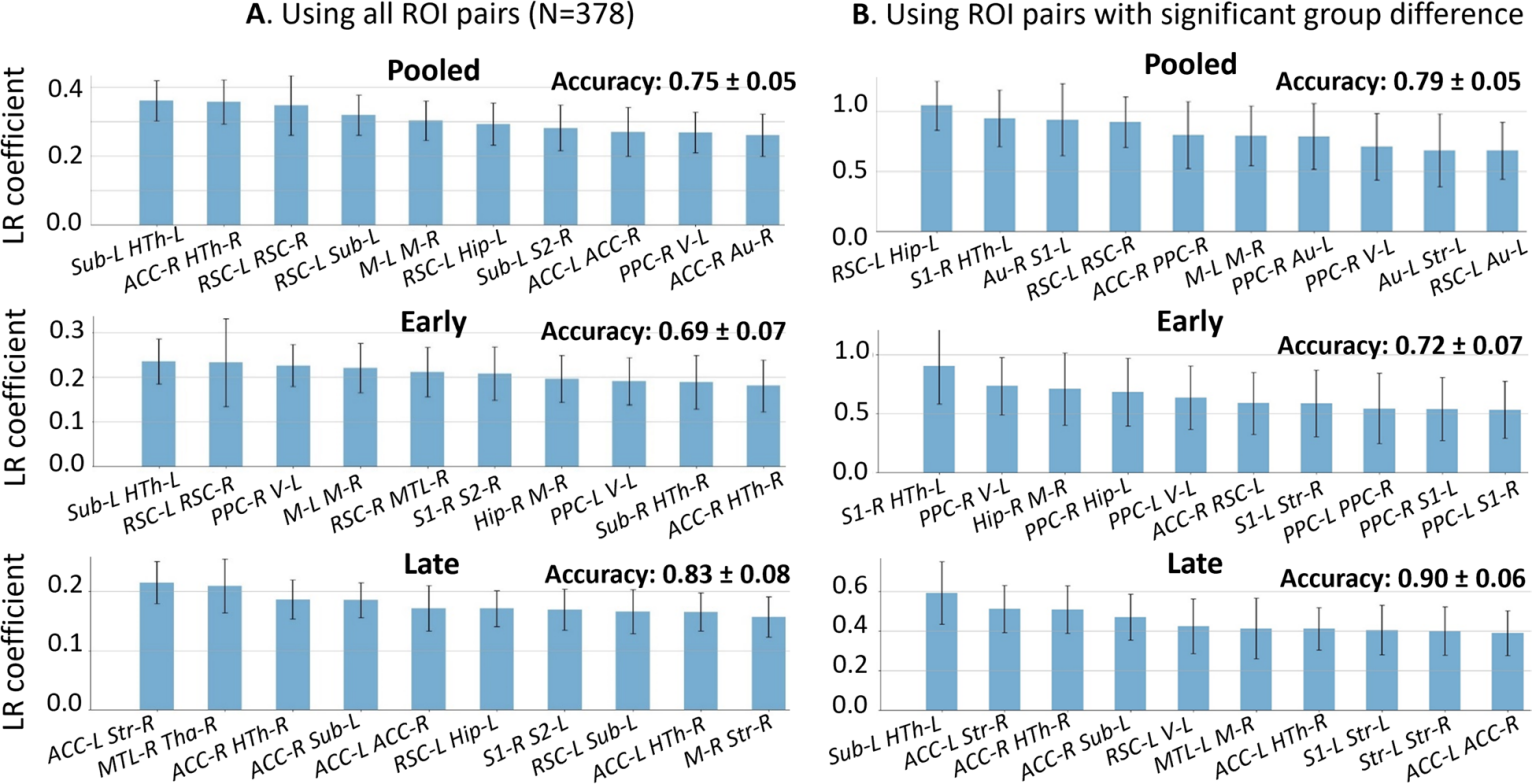
**A** Top ten features (out of 378) and their importance in terms of absolute LR coefficient in rat classification on the FC dataset (mean ± standard deviation, averaged over 1000 repetitions). Each feature is an edge. The most relevant edges that discriminate between CTL and STZ rats involve ACC, hypothalamus (HTh), RSC, hippocampus (Hip), and subiculum (Sub). **B** Only connections surviving the NBS significance test were selected as features for classification (top 10 displayed). Classification accuracy was improved from 0.75 to 0.79 for pooled, 0.69 to 0.72 for early, and 0.83 to 0.90 for Late dataset by this feature pre-selection. Higher classification accuracy in late dataset is consistent with the advanced stage of disease and more marked differences between STZ and CTL

When using all connections as features (*N* = 378), prediction accuracy on the pooled, early, and late datasets was 0.75, 0.69, and 0.83, respectively. The most relevant edges involved the ACC, hypothalamus, RSC, hippocampus, and subiculum as nodes (Fig. 5A), in agreement with edges found as significantly different between groups in the NBS analysis (Fig. 4). When only significant edges from the NBS analysis were selected as a reduced list of features for classification (*N* = 49, 38, and 71 features in the pooled, early, and late datasets, respectively), the classification accuracy improved to 0.79 for pooled, 0.72 for early, and 0.90 for late datasets (Fig. 5B). Improved accuracy was not strictly related to feature reduction: reducing features using PCA deteriorated classification accuracy (data not shown). Notably, the highest classification accuracy was found on the late dataset which is consistent with the advanced stage of disease and more marked differences between STZ and CTL.

However, the top 10 edges with the highest feature importance in the first classification (all features, Fig. 5A) did not overlap strongly with that from the second classification (reduced features, Fig. 5B) perhaps due to the small sample size. The nodes involved in the top 10 edges did however overlap strongly between the two classifications.

**Fig. 6:**
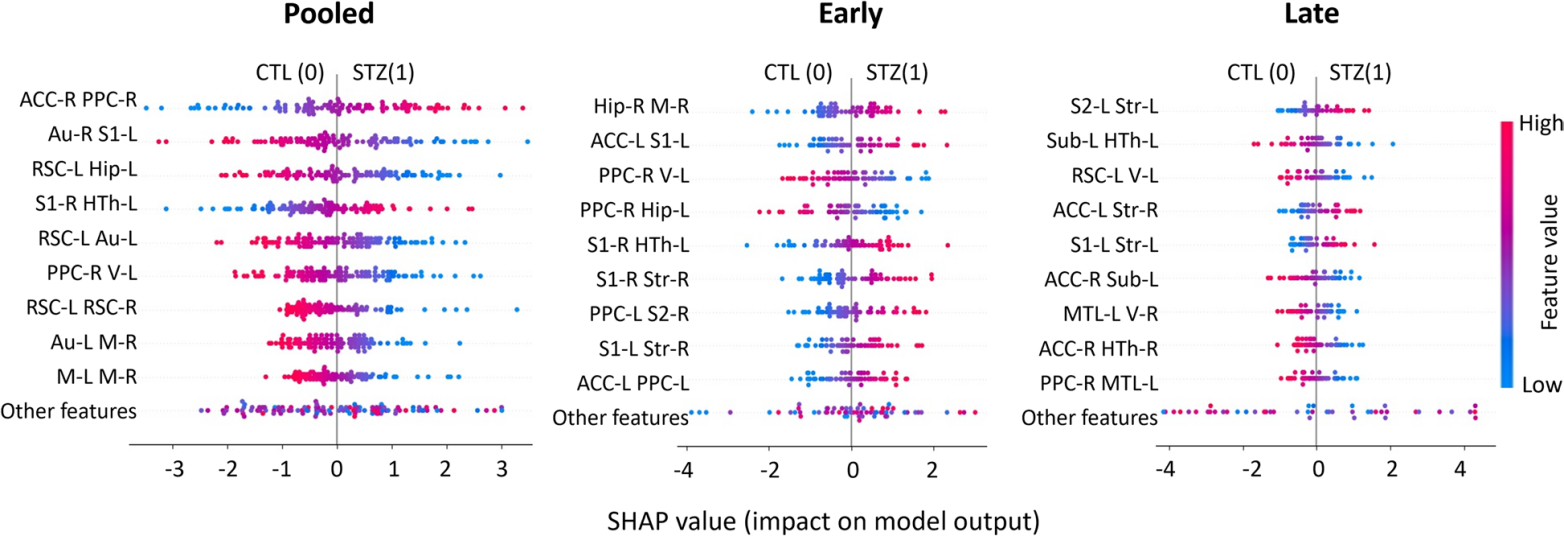
Exemplary SHAP summary plots for the three datasets (pooled, early, and late) based on the model using FC significant connections as features. The summary plot combines feature importance with feature effects. Each point on the summary plot is a SHAP value for a feature and an instance. The position on the *y*-axis is determined by the feature and on the *x*-axis by the SHAP value. The color represents the value of the feature from low (blue) to high (red). The features are ordered according to their importance (top 9 displayed). Positive SHAP values lead the model to predict 1 (STZ) while negative ones lead the model to predict 0 (CTL)

Figure 6 displays SHAP plots for each instance of the most important features used to classify STZ and CTL subjects in each of the three datasets. Top features were generally consistent with those from LR in Fig. 5A. Distribution of values for each feature (edge) in the STZ and CTL groups also agreed with the group difference test in the form of graph networks (Fig. 4). For example, in the early timepoints, both methods revealed the STZ group had stronger connectivity between the right hippocampus and motor cortex, left ACC and S1, left hypothalamus and right S1, and reduced connectivity between right PPC and left visual cortex, as well as right PPC and left hippocampus. In the late timepoints, the STZ group had increased connectivity between left S2 and striatum, left ACC and right striatum, left S1 and striatum, and weaker connectivity between left subiculum and hypothalamus, left RSC, and visual cortex. Overall, the distribution of SHAP values demonstrated that the STZ group had hyperconnectivity in the early timepoint but hypoconnectivity in the late timepoint, which confirmed the findings in the previous study [97].

### Microstructure-based classification

**Fig. 7:**
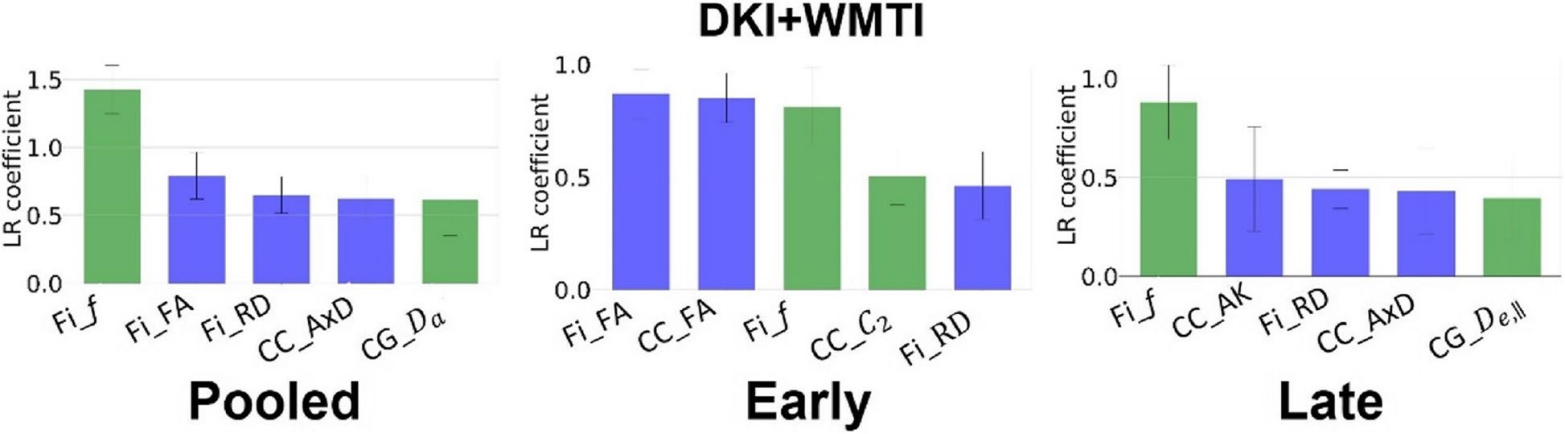
Feature importance and test classification accuracy using different microstructure metrics (mean ± std over 1000 repetitions). Displayed are the top 5 most import features on the three datasets using DKI metrics (blue) and WMTI parameters (green) altogether. fi, fimbria; cc, corpus callosum; cg, cingulum; FA, fractional anisotropy; AD/RD, axial/radial diffusivity; AK, axial kurtosis; *f,* axonal density; *D*_a_, intra-axonal diffusivity; D_e,||_, extra-axonal parallel diffusivity; c_2_: orientation dispersion

For classification based on WM microstructure features, the mean test accuracy and top features with the highest importance are displayed in Fig. 7. When using the combined diffusion metrics (DKI + WMTI) as features, the FA in fimbria and corpus callosum stood out as the best discriminating features in the early timepoints while the axonal density (*f*) of the WMTI-Watson model in the fimbria was the most important feature in the late timepoints as well as in the pooled data. Overall, the fimbria microstructure was the best discriminator between groups. FA was sensitive to early changes in STZ rats, which drove the DKI-based model to achieve better classification accuracy than the WMTI-based model at the Early timepoint (Table 1). While the accuracy of the DKI-based classifier decreased significantly at the Late timepoint, the accuracy of the WMTI-based classifier remained stable across time. The classifier built on combined DKI + WMTI metrics obtained the highest accuracy in the early stage and similar accuracy in the late.

**Table 1:** The accuracy of classification on the three datasets and cross predictions in the different cases of employing separate and joint features. The last column is the total data size in the pooled dataset. The FC dataset has a larger sample size because each rat subject had two fMRI scans for each experiment. Dimension reduction using PCA did not improve the classification accuracy in most cases except for FC-based classification on the Early dataset and the late-to-early cross prediction where the new accuracies and the optimal numbers of PCA components were indicated. In FC-based classification, only connections with significant group differences were retained except for cross predictions where all FC connections were used. For pooled, early, and late timepoints, the classification accuracy is the average over 1000 random data splits into 70% training and 30% testing. For early-to-late, the training set was all early datasets and the test set all late datasets (and vice versa for late-to-early)

Features	Pooled	Early	Late	Early-to-late	Late-to-early	Sample size
FC	0.79 ± 0.05	0.72 ± 0.07 *(0.75, PCA* = *10)*	0.90 ± 0.06	0.69	0.61 *(0.7, PCA* = *15)*	*N* = 162
DKI	0.81 ± 0.07	0.87 ± 0.09	0.79 ± 0.10	0.74	0.76	*N* = 83
WMTI	0.84 ± 0.07	0.81 ± 0.10	0.82 ± 0.09	0.87	0.78	
DKI + WMTI	0.84 ± 0.06	0.88 ± 0.10	0.81 ± 0.09	0.82	0.78	
FC + DKI + WMTI	0.82 ± 0.08	0.77 ± 0.09	0.87 ± 0.09	0.79	0.76	*N* = 79
Ensemble (FC, DKI, WMTI)	0.85 ± 0.07	0.85 ± 0.09	0.86 ± 0.09	0.82	0.73	

**Fig. 8:**
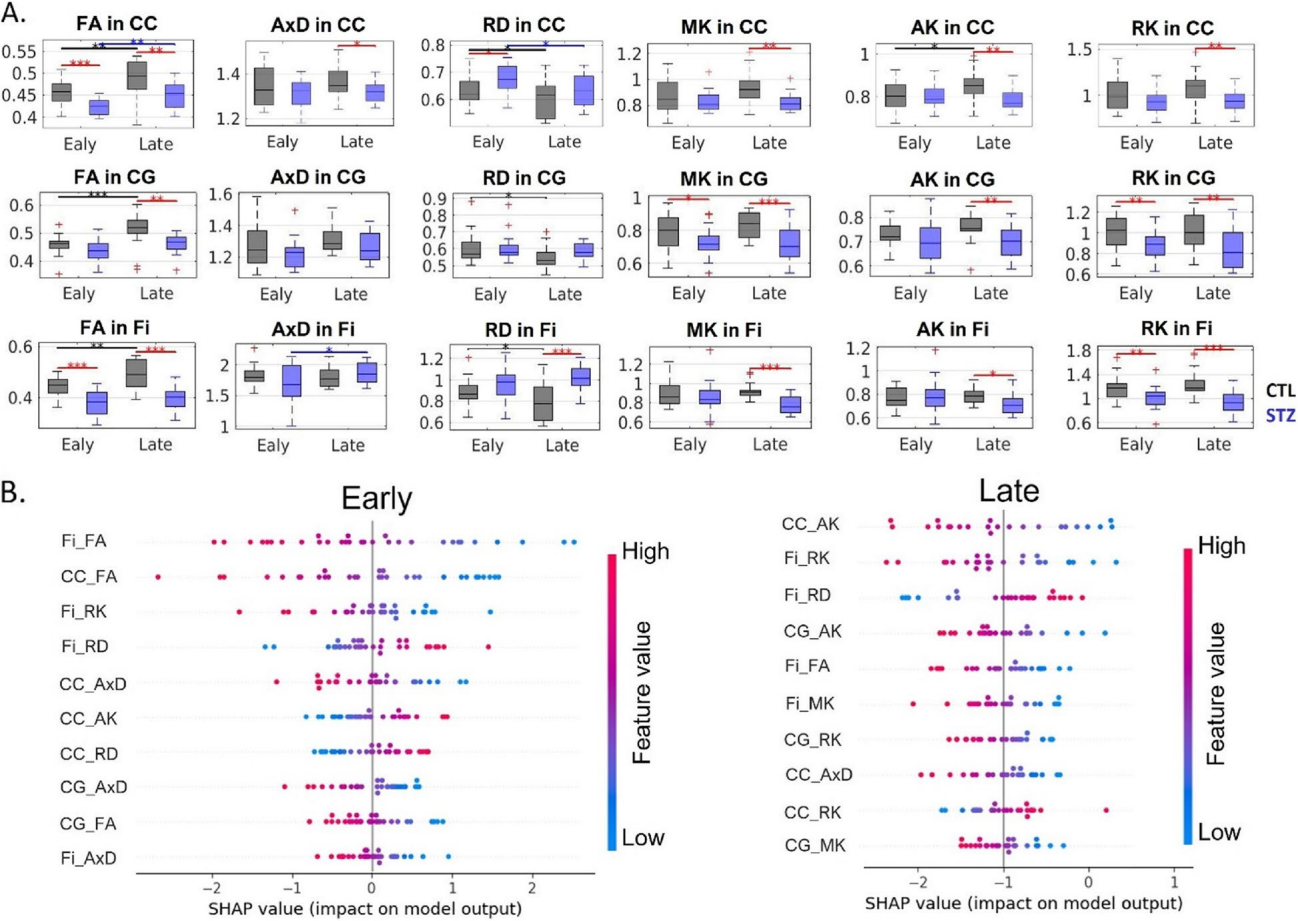
**A** DKI estimates in three white matter ROIs (top row: corpus callosum (CC), middle row: cingulum (CG), and bottom row: fimbria of the hippocampus (Fi)). FA, fractional anisotropy; AxD/RD, axial/radial diffusivity; MK/AK/RK, mean/axial/radial kurtosis. Two-tailed *t*-test for inter-group comparison (red bars) and one-way ANOVA with Tukey-Cramer correction for within-group comparison across time (black and blue bars). ∗ : *p* < 0.05, ∗  ∗ : *p* < 0.01, ∗  ∗  ∗ : *p* < 0.001. + : outlier values (but not excluded from the analysis). **B** SHAP summary plots combining feature importance with feature effects based on DKI estimates. The position on the *y*-axis is determined by the feature and on the *x*-axis by the SHAP value. The color represents the value of the feature from low (blue) to high (red). The features are ordered according to their importance (top 10 displayed). Positive SHAP values lead the model to predict 1 (STZ) while negative ones lead the model to predict 0 (CTL)

**Fig. 9:**
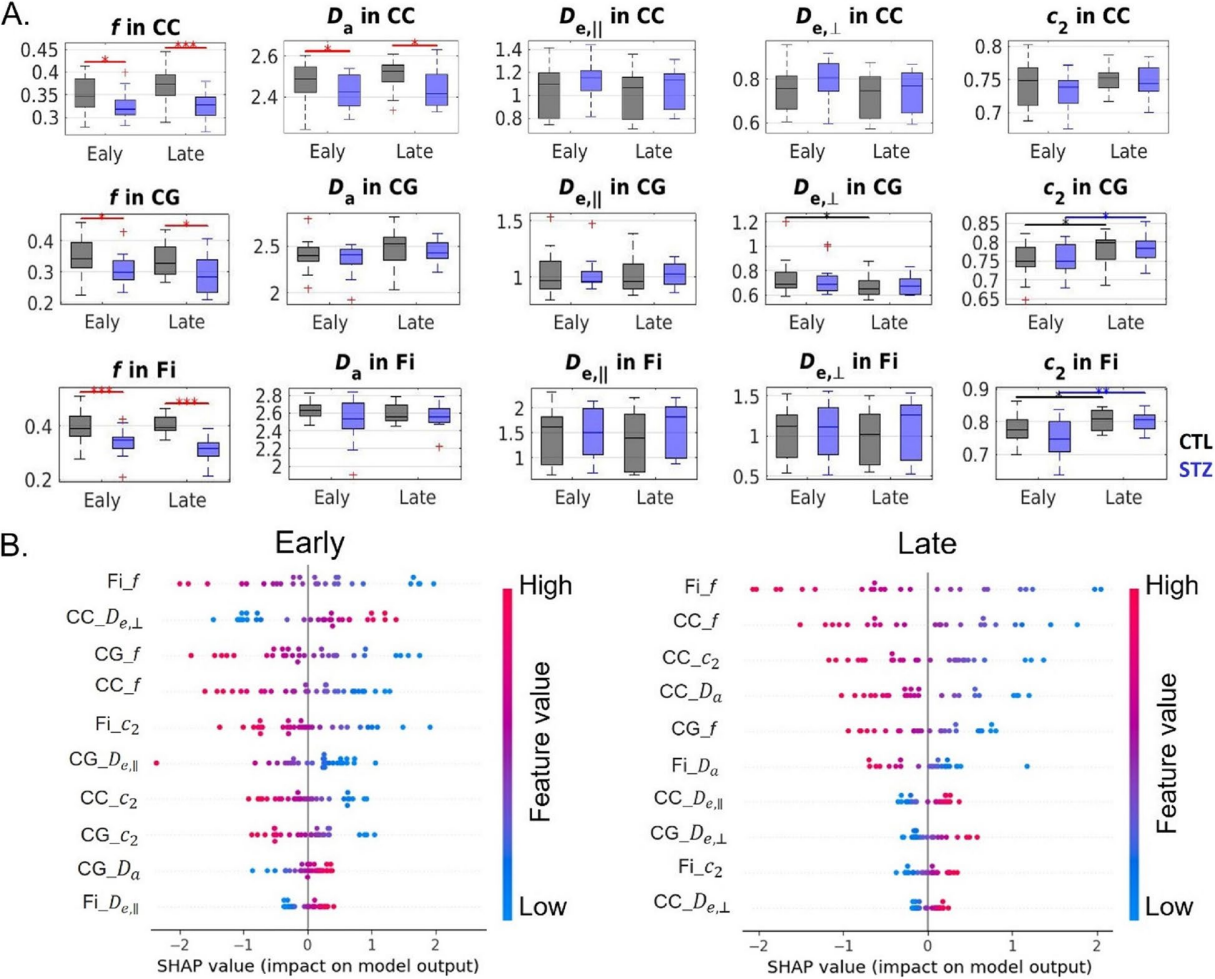
**A** WMTI-Watson model estimates in three white matter ROIs (top row: corpus callosum (CC), middle row: cingulum (CG), and bottom row: fimbria of the hippocampus (Fi)). Two-tailed *t*-test for inter-group comparison (red bars) and one-way ANOVA with Tukey-Cramer correction for within-group comparison across time (black and blue bars). ∗ : *p* < 0.05, ∗  ∗ :* p* < 0.01, ∗  ∗  ∗ : *p* < 0.001. + : outlier values (but not excluded from the analysis). **B** SHAP summary plots combining feature importance with feature effects based on WMTI-Watson model estimates. The position on the *y*-axis is determined by the feature and on the *x*-axis by the SHAP value. The color represents the value of the feature from low (blue) to high (red). The features are ordered according to their importance (top 10 displayed). Positive SHAP values lead the model to predict 1 (STZ) while negative ones lead the model to predict 0 (CTL)

Figures 8 and 9 report the SHAP value for each feature and each prediction of the LR classifiers based on DKI and WMTI parameters. As for FC, a high degree of consistency was found between metrics with high SHAP values and those displaying group differences between STZ and CTL rats. Specifically, lower FA and higher RD in corpus callosum, and lower FA and RK in fimbria were major drivers of STZ difference to CTL in the early timepoints. In the late timepoints, reduced AxD and AK in the corpus callosum; decreased MK, AK, and RK in the cingulum; and decreased FA, MK, and RK, as well as increased RD in fimbria, were found to be the most prominent features distinguishing the STZ group from the CTL. WMTI-Watson parameters provided us with more specificity to differences between STZ and CTL groups. In both early and late timepoints, the white mater of STZ rats was characterized by lower intra-axonal diffusivity (*D*_a_) in CC, indicating intra-axonal damage, and lower axonal water fraction (*f*) in CC, cingulum, and fimbria, indicating demyelination and axonal loss.

### Combining the FC and microstructure features

After combining the FC and microstructure features, the number of total rat subjects in the pooled dataset was reduced from 83 to 79 (Table 1) due to the absence of either fMRI or diffusion MRI data for four datasets. The two combination methods — concatenation vs ensemble — had similar mean classification accuracy on the Late dataset, but the ensemble method achieved much higher accuracy (10% improvement) on the Early dataset, and slightly better accuracy on the Pooled dataset. Overall, neither combined classifier outperformed single classifiers at a given timepoint: best early classification accuracy was achieved by DKI + WMTI and best late classification accuracy by FC.

Looking at the cross-prediction performance for all classifiers, the WMTI classifier trained on the early dataset obtained an outstanding accuracy on the late dataset, which was even higher than that of the classifier trained on the late data (0.87 vs 0.82). In addition, both cross-prediction classifiers (late-to-early and early-to-late) based on WMTI features had better accuracy than those based on DKI features or combined DKI + WMTI features. The FC classifier however had poor cross-prediction performance. This may indicate inter-group differences in FC evolved significantly from the early stage towards the late stage, which was consistent with early hyperconnectivity and late hypoconnectivity in STZ (Fig. 3). The combination methods had moderate performance both in early-to-late and late-to-early predictions. With the exception of DKI, all classifiers had higher accuracy in early-to-late prediction than late-to-early.

**Fig. 10:**
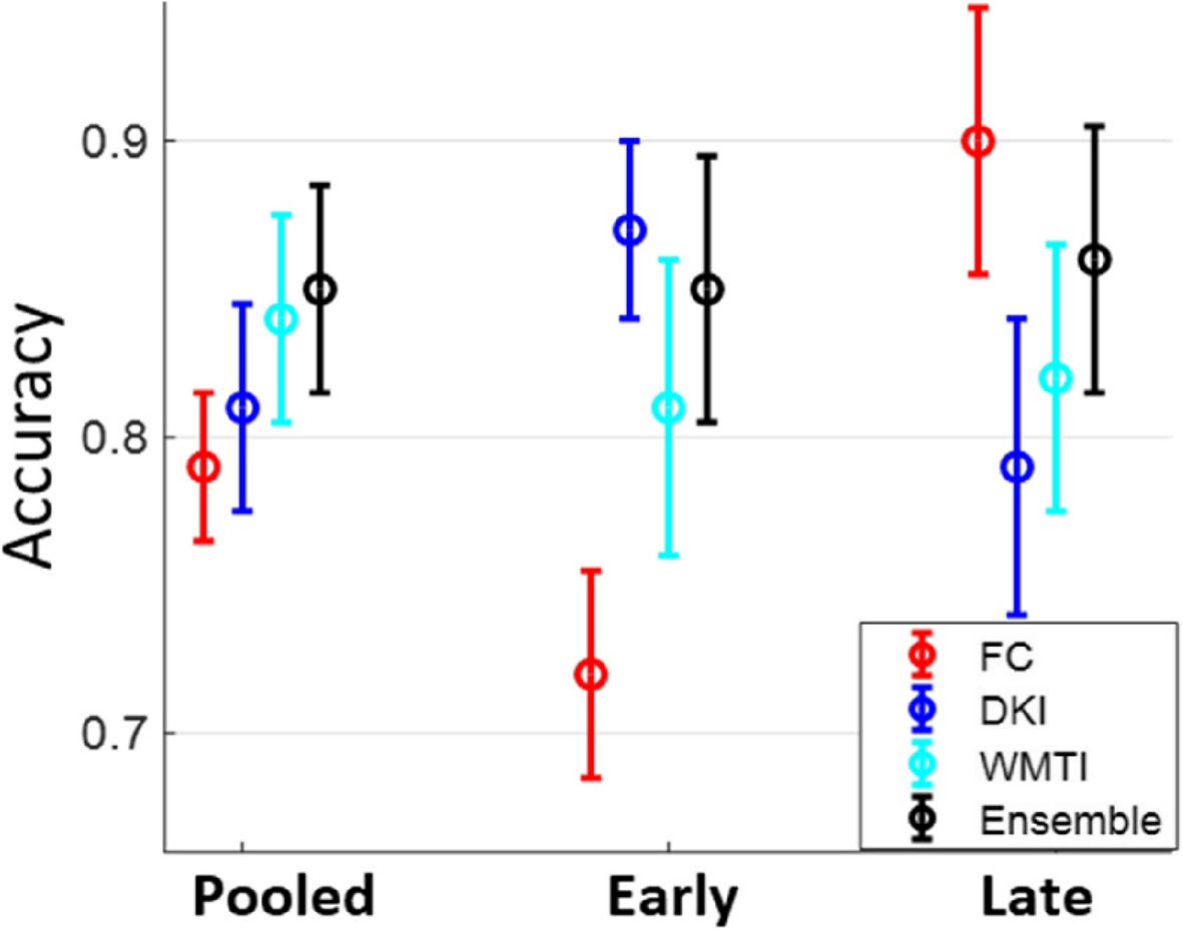
A summary plot of classification accuracy on the three datasets (pooled, early, and late) for each individual classifier and the ensemble classifier

Based on Table 1, a summary plot of classification accuracy based on either FC or microstructure metrics as well as the ensemble method on the three datasets (pooled, early, and late) is shown in Fig. 10. On the Pooled data, the ensemble method achieved the highest overall accuracy among classifiers, which revealed that the best strategy was to combine all three types of features (FC, DKI, WMTI) in an ensemble-learning way. However, at the early timepoint, classification based on WM microstructure, especially DKI, provided substantially higher accuracy than FC-based classification while at the late timepoint, the FC-based classification significantly outperformed the microstructure-based classification. One possible explanation is that WM microstructure damage happens earlier than alterations in functional connectivity in the STZ group. However, this assumption needs to be further validated in human Alzheimer’s studies.

## Discussion

The classification of individuals with AD or mild cognitive impairment from healthy controls using MRI-based features and ML has been increasingly proposed. Several studies have reported promising results of employing resting-state FC as major features to this end [45, 47, 58, 76, 103, 105, 112]. A few studies have also proposed using WM DTI-based features such as FA and MD for the classification of AD subjects [11, 28, 55, 70].

Indeed, longitudinal studies of MCI and AD populations compared to healthy controls have revealed distinct WM degeneration patterns, as characterized using DTI, between patient and healthy populations. Decreased FA and increased MD over the course of one year were reported in the hippocampal cingulum of the AD group [72], both in the cingulum and fornix in an MCI and AD cohort [79], and genu of the corpus callosum in an MCI cohort [96]. Rates of WM structural decline were also faster in subjects initially enrolled in the preclinical phase of MCI and AD and who eventually developed dementia, mainly evidenced by a decrease in FA in the right inferior fronto-occipital fasciculus and splenium of corpus callosum [90]. Another study reported higher rates of change in FA and RD in the splenium of the corpus callosum, posterior cingulum, and left superior temporal region over the course of one year in AD [1]. Cross-sectional studies where FDG-PET and dMRI were available jointly suggested strong correlations between hypometabolism and altered DTI metrics in the hippocampus or posterior cingulate in early AD and amnestic MCI patients, with higher DTI sensitivity to early disease [107, 115].

Similarly, longitudinal fMRI studies showed early stages of the disease are characterized by hyperconnectivity of certain brain networks, while follow-up in time inevitably leads to decreased connectivity throughout the brain. For example, subjects with an initial Clinical Dementia Rating (CDR) of 0.5 displayed reduced activity in the right hippocampus after 2 years, while those CDR of 0 did not, while the rate of decline correlated positively with high hippocampal activity at baseline, further supporting the non-monotonic pattern of initial hippocampus hyperconnectivity followed by hypoconnectivity as dementia progresses [80]. Similarly, initial hyperconnectivity of the anterior and ventral DMN transitioned to hypoconnectivity at follow-up in AD patients [22]. In cross-sectional studies of simultaneous FDG-PET/fMRI, the spatial brain patterns of hypoconnectivity and hypometabolism overlapped only partially, while each maintaining the good predictive value of cognitive decline [71, 111].

However, multi-model longitudinal studies in humans over a significant time span are extremely challenging to achieve. To our knowledge, no study reported longitudinal metabolic, microstructural, and functional connectivity changes jointly, allthemore using advanced diffusion metrics beyond DTI. Furthermore, the value of WM microstructure and FC features for subject classification and cross timepoint prediction has not been evaluated. A recent cross-sectional study has evaluated the value of amyloid, tau, glucose hypometabolism, and structural atrophy in classifying MCI and AD patients, with amyloid and tau being better predictors of MCI and early AD, while glucose hypometabolism and atrophy were better predictors of later AD [41].

Animal models are very well suited to perform comprehensive longitudinal studies over a time period that covers a broad range of pathology evolution. The icv-STZ rat model induces impaired brain glucose metabolism, which is an excellent biomarker for disentangling AD from other forms of dementia. Rats further exhibit several features of Alzheimer’s at multiple levels: pathological (tau, amyloid, neuronal loss, atrophy), behavioral (short-term memory impairment), and neuroimaging (same trends in diffusion and rs-fMRI metrics as in humans).

To our knowledge, this study is the first one attempting to evaluate FC and WM microstructure features separately as well as their combination in a ML-based classification context. Furthermore, apart from the conventional DTI metrics, features based on more advanced DKI metrics and especially on biophysical models were also assessed in this study.

In support of the empirical relevance of the icv-STZ model for sporadic AD, the most important discriminating features in FC and WM integrity aligned with brain regions and WM tracts affected in human sporadic AD itself. Discriminating FC connections involved regions of the default mode network such as the hippocampus, cingulate, and posterior parietal cortex [2, 15, 97], as well as the hypothalamus which is responsible for recruiting alternative sources of energy to glucose, such as ketone bodies, in response to impaired brain glucose metabolism by the STZ [17, 33, 36, 64, 106]. Many FC connections with top feature importance in the Early stage also involved the visual and motor cortices, areas that are related to non-cognitive manifestations such as vision and motor decline and have been reported to precede the cognitive deficits in humans [14, 27, 44, 48, 73, 74, 102]. For classification based on WM integrity, microstructural features in the fimbria of the hippocampus played the most important role in distinguishing STZ rats, which was consistent with the fact that hippocampus is especially vulnerable to AD [77, 89] and to the icv-STZ rat model of AD [4, 92].

Our results show DKI brings valuable complementary information to DTI for classification purposes, and the WM model narrows down the identification of microstructure changes to intra-axonal damage, demyelination, and axonal loss. This is in line with the expectation from biophysical models to increase specificity to microstructure features over signal representation metrics such as DTI or DKI [49, 50, 78]. Going forward, the acquisition of multi-shell diffusion MRI data (at least two non-zero *b*-values, e.g., *b* = 1000 and 2500 s/mm^2^) in clinical studies of dementia or other brain diseases is highly recommended to enable the estimation of DKI metrics brain-wide, and of WM microstructure features using the WMTI-Watson model, for which analysis code is readily available [24]. WMTI metrics were arguably the most stable features in discriminating STZ and CTL subjects compared to the DKI- and FC-based features, as evidenced in the cross-timepoint prediction accuracy (> 0.80). This might indicate the possibility of early screening and prognosis of AD in clinical applications using WM microstructure features derived from the WMTI-Watson model of diffusion. In other words, subjects with early WM alterations at high risk of developing further neurodegeneration might be identified and receive intervention when they are still in the early stage [99].

When using FC to classify STZ/CTL rats, only choosing connections significantly different between groups (using NBS) as features naturally improved mean classification accuracy. When translating our classification approach to discriminate AD patients from healthy controls, FC edges identified as driving group differences between diagnosed AD patients and controls could be used as features for classification in future diagnostic-blind studies, or to discriminate prospective AD patients from controls.

From the perspective of pathological progression and biomarker timeline within the course of the disease, microstructure-based features achieved better performance than FC in the early timepoint as well as for cross-timepoint predictions. Performance in the early timepoint suggests that WM degeneration in the STZ group could happen earlier than FC breakdown. Similar findings have been reported by human studies in subjective cognitive impairment as well as AD [6, 70, 82]. Performance in the cross-timepoint prediction suggests that microstructure degeneration is relatively consistent across time. In contrast, the pattern in FC metrics was non-monotonic and shifted from early hyperconnectivity to late hypoconnectivity in the STZ rats, as also previously reported in human studies [26]. However, more data are required to fully validate these hypotheses, especially in humans.

Nevertheless, the best overall strategy for STZ vs CTL classification was aggregating the three individual classifiers using ensemble learning. Not only was the ensemble classification more accurate on the pooled dataset (0.85) than any of the individual classifiers, but it also maintained a high level of accuracy at each of the separate timepoints. This demonstrated that microstructural and functional information can be complementary and have their unique value in identifying STZ rats, and possibly mild cognitive impairment and early AD.

As to limitations, first, this study is based on a relatively small dataset with 24 rats followed across four timepoints. Second, we only used male rats, which was based on practical reasons. As female rats are more resistant than males to STZ-induced alterations [10, 35, 86] and hormonal modulation plays an important role in females, future studies should consider rats of both sexes. Third, in FC-based classification, each connection (ROI pair) was treated as an individual feature leading to the loss of the topological information among them. For future studies, graph neural networks can be used to replace LR for the FC-based classification [65, 114] since they consider the functional network as a whole thus better preserving spatial information. However, this will also require more advanced explainability methods to interpret the classification results [108, 113]. Finally, no amyloid or tau information was available for these rats in vivo. However, histological stainings performed after sacrifice at 21 weeks revealed amyloid plaques and neurofibrillary tangles in icv-STZ brains, as reported in our previously published study [97]  as well as other studies of this animal model [30, 60, 91].

## Conclusions

Our work examined potential discriminators of Alzheimer’s disease in the icv-STZ rat model using functional connectivity and WM microstructure features. For the first time, we evaluated those two types of MRI-based features separately as well as in combination, in a context of ML-based classification. WM microstructure features achieved higher classification accuracy in the early timepoints of neurodegeneration, and FC in the later timepoints, suggesting structural damage precedes functional damage. Combining all the FC and microstructure metrics in an ensemble way was the best strategy to discriminate between STZ and CTL rats, with a consistent accuracy over time above 0.85. However, for cross-time prediction, WMTI model features yielded the highest accuracy from early-to-late timepoints and vice versa, possibly thanks to the more specific metrics they capture from the microstructure, that project well across timepoints. Foreseeably in human datasets, the best microstructure (or ensemble microstructure + FC) classification features would be extracted from late timepoints with known subject diagnosis (e.g., healthy vs AD), the ML model trained on late timepoint datasets of those reduced features, and then applied to early timepoint populations to aid early diagnosis and prediction of disease evolution.

### Supplementary Information

**Additional file 1: Figure S1.** Example of rs-fMRI images of 8 coronal slices (A), matching anatomical MR images (B) and atlas-based anatomical labels registered to the fMRI images (C). (Image taken from https://www.frontiersin.org/articles/10.3389/fnins.2021.602170/full). **Table S1.** A list of 28 atlas-defined regions of interest (ROIs, 14 per hemisphere) for the fMRI data. The ROIs were regrouped based on the original labels of the Waxholm Space (WHS) Atlas of the rat brain (https://www.nitrc.org/projects/whs-sd-atlas).

## Data Availability

The datasets generated and/or analyzed during the current study are available in the OpenNeuro repository, https://openneuro.org/datasets/ds003520/versions/1.0.2 (resting-state fMRI, cohort 1, *N* = 17 rats) and https://openneuro.org/datasets/ds004441 (diffusion MRI, cohorts 1 + 2, *N* = 24 rats).

## Abbreviations

ACC: Anterior cingulate cortex
AD: Alzheimer’s disease
AxD: Axial diffusivity
AK: Axial kurtosis
CC: Corpus callosum
CG: Cingulum
CTL: Control
DKI: Diffusion kurtosis imaging
DMN: Default mode network
DTI: Diffusion tensor imaging
FA: Fractional anisotropy
FC: Functional connectivity
Fi: Fimbria
fMRI: Functional MRI
icv: Intracerebroventricular
LR: Logistic regression
MD: Mean diffusivity
MK: Mean kurtosis
ML: Machine learning
MRI: Magnetic resonance imaging
PCA: Principal component analysis
PPC: Posterior parietal cortex
RD: Radial diffusivity
RK: Radial kurtosis
ROI: Region of interest
RSC: Retrosplenial cortex
S1: Primary somatosensory cortex
S2: Secondary somatosensory cortex
STZ: Streptozotocin
WM: White matter
WMTI-Watson: White Matter Tract Integrity Watson model
