# Supplementary material for: Subject classification and cross-time prediction based on functional connectivity and white matter microstructure features in a rat model of Alzheimer’s using machine learning
Source: Alzheimers Res Ther. 2023 Nov 7;15:193. doi: 10.1186/s13195-023-01328-0 (PMC10629161; doi:10.1186/s13195-023-01328-0)
Supplement: Supplementary file 1 — Additional file 1: Figure S1. Example of rs-fMRI images of 8 coronal slices (A), matching anatomical MR images (B) and atlas-based anatomical labels registered to the fMRI images (C). (Image taken from https://www.frontiersin.org/articles/10.3389/fnins.2021.602170/full). Table S1. A list of 28 atlas-defined regions of interest (ROIs, 14 per hemisphere) for the fMRI data. The ROIs were regrouped based on the original labels of the Waxholm Space (WHS) Atlas of the rat brain (https://www.nitrc.org/projects/whs-sd-atlas). [file 13195_2023_1328_MOESM1_ESM.pdf]

Subject classification and cross-time prediction based on functional connectivity and white matter microstructure features in a rat model of Alzheimer's using machine learning

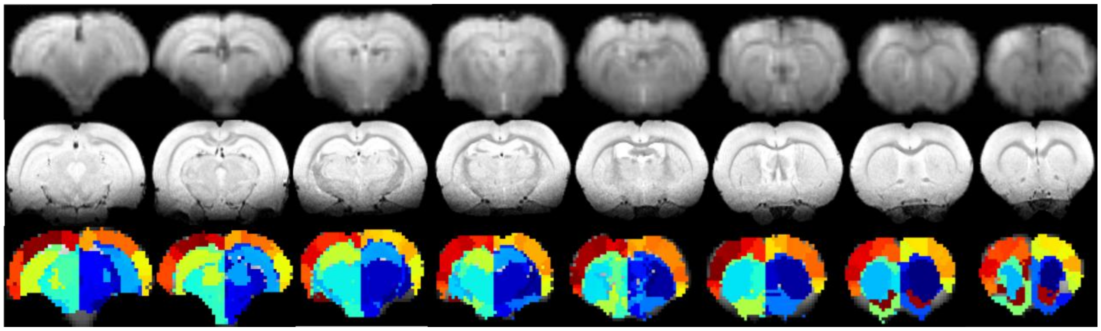

Figure S1 Example of rs-fMRI images of 8 coronal slices (A), matching anatomical MR images (B) and atlas-based anatomical labels registered to the fMRI images (C). (Image taken from https://www.frontiersin.org/articles/10.3389/fnins.2021.602170/full)

Table S1 A list of 28 atlas-defined regions of interest (ROIs, 14 per hemisphere) for the fMRI data. The ROIs were regrouped based on the original labels of the Waxholm Space (WHS) Atlas of the rat brain (https://www.nitrc.org/projects/whs-sd-atlas).

**ROIs**	**Names**	**ROI Labels (Right)**	**ROI Labels (Left)**	**Original WHS Labels (Right)**	**Original WHS Labels (Left)**
ACC	cingulate cortex	2	1	164,165	212,213
RSC	retrosplenial cortex	4	3	183,184,185	231,232,233
PPC	posterior parietal cortex	6	5	174,178,180,181,182	222,226,228,229,230
MTL	temporal association cortex	8	7	179,197,170,167,168, 177,204	227, 245, 218, 215, 216, 225, 252
Hip	hippocampus	10	9	73,74,75,76	151,152,153,154
Sub	subiculum	12	11	70	148
AU	auditory cortex	14	13	161,162,163	209,210,211
V	visual cortex	16	15	198:203	246:251
S1	primary somatosensory cortex	18	17	186:195	234:243
S2	secondary somatosensory cortex	20	19	196	244
M	motor cortex	22	21	175,176	223,224
CPu	striatum	24	23	8	86
Tha	thalamus	26	25	17	95
HTh	hypothalamus	28	27	26	104
